# Citrate Mediated Europium-Based Detection of Oxytetracycline in Citrus Tissues

**DOI:** 10.3390/antibiotics10050566

**Published:** 2021-05-12

**Authors:** Faraj Hijaz, Yasser Nehela, Ozgur Batuman, Nabil Killiny

**Affiliations:** 1 Citrus Research and Education Center, Department of Plant Pathology, IFAS, University of Florida, 700 Experiment Station Road, Lake Alfred, FL 33850, USA; fhijaz@ufl.edu (F.H.); yasser.nehela@ufl.edu (Y.N.); 2 Department of Agricultural Botany, Faculty of Agriculture, Tanta University, Tanta 31512, Egypt; 3 Southwest Florida Research & Education Center, Department of Plant Pathology, IFAS, University of Florida, 2685 State Road 29 North, Immokalee, FL 34142, USA; obatuman@ufl.edu

**Keywords:** oxytetracycline, Huanglongbing, europium, citrate, antibiotic, citrus

## Abstract

Oxytetracycline (OTC) and streptomycin have been used for the control of several plant diseases and were recently permitted for the control of citrus greening disease, Huanglongbing. Consequently, sensitive and reliable methods are highly needed for the detection of OTC in citrus tissues. Herein, we studied the replacement of cetyltrimethylammonium chloride (CTAC) by citrate (Cit) as a sensitizing agent for the analysis of OTC in citrus tissues using the recently established europium (Eu) method. In addition, we determined the optimal conditions for the formation of the Eu-OTC-Cit ternary complex in tris buffer. Our results showed that the plant matrix significantly decreased the fluorescence intensity of the Eu-OTC-Cit complex even after the replacement of CTAC. Our investigations showed that phenols such as gallic acid degrade slowly at high pH and their degradation was enhanced in the presence of the (Eu^+3^) cation. To reduce the plant matrix interference, the sample extract was cleaned using solid-phase extraction (SPE). The OTC recoveries from spiked healthy and *Candidatus* Liberibacter asiaticus (*C*Las)-infected trees were 91.4 ± 7.8% and 82.4 ± 3.9%, respectively. We also used the citrate method to determine the level of OTC in trunk-injected trees. The level of OTC as measured using the Eu-OTC-Cit complex (117.5 ± 20.3 µg g^−1^ fresh weight “FWT”) was similar to that measured using Eu-OTC-CTAC complex (97.5 ± 14 µg g^−1^ FWT). In addition, we were able to visualize the OTC in citrus leaf extract, under ultraviolet light (400 nm), after it was cleaned with the SPE. Our study showed that the citrate can be successfully used to replace the harmful CTAC surfactant, which could also react with phenols.

## 1. Introduction

Oxytetracycline (OTC) is a tetracycline antibiotic with a wide range of antibacterial activity and high potency [1]. OTC has been approved for the treatment of humans and animals including cattle and poultry [1]. Besides, OTC has been used for the control of several plant diseases including the yellow diseases in the coconut palm trees, spot disease in peaches, and several bacterial pathogens on vegetables [2]. Recently, OTC and streptomycin were also permitted for the control of citrus greening disease, Huanglongbing [3]. This decision was issued after the tremendous loss in the citrus industry in the last decade. The idea for using antibiotics for the control of Huanglongbing was initiated in the 1970s after it was discovered that it was caused by a bacterial pathogen [4]. Huanglongbing is believed to be caused by the *Candidatus* Liberibacter asiaticus (*C*Las), which is vectored by *Diaphorina citri*. Huanglongbing is currently considered the most destructive citrus disease in the US, which causes a significant loss of production and rapid tree death.

Previous results showed that several antibiotics, including penicillin, tetracycline, and ampicillin, were effective against the *C*Las pathogen [5]. A recent study also showed that trunk injection of OTC in ‘Hamlin’ sweet orange trees significantly reduced (>99%) the *C*Las titer twenty-eight days post-injection [6]. Trunk injection of OTC also increased fruit yield and slightly decreased juice acidity [6]. In a previous study, we showed that OTC was taken by citrus seedlings after root drench and trunk application [7]. High levels of OTC were detected in the leaf (~15 µg g^−1^ FWT), phloem (~250 µg g^−1^ FWT), and xylem (~180 µg g^−1^ FWT) of citrus seedlings after stem delivery [7]. The presence of OTC in the phloem, where the *C*Las inhabits, indicated that it could be efficient against this important pathogen. Besides, we studied the translocation of OTC in girdled and non-girdled seedlings and trees [8]. Our results showed that girdling did not affect the movement of OTC, indicating that the xylem was the main route for OTC movement [8]. The presence of OTC in the phloem beyond the girdling indicated that OTC could be translocated from the xylem to the phloem during acropetal movement [8]. Recently, we studied the uptake of OTC by citrus trees in the field after trunk and foliar application [9]. Our results showed that foliar spray of OTC was less effective than trunk injection [9]. Higher levels of OTC (~5 µg g^−1^ FWT) were found in trunk-injected trees compared with those treated using the foliar application (~0.1 µg g^−1^ FWT) [9]. The *C*Las titer was substantially reduced after trunk injection, whereas no effect was observed after the foliar application [9]. Our results also demonstrated that the use of adjuvants did not enhance uptake of OTC by citrus leaves [9].

Although many methods have been established for the detection of OTC including liquid chromatography and several colorimetric methods, the enzyme-linked immunosorbent assay (ELISA) is considered the most preferred method [10]. The ELISA method is fast, simple, sensitive, and can be used to examine many samples at the same time [10]. In our previous studies, we used the ELISA kit to study the uptake and translocation of OTC in citrus plants after root drench, trunk injection, and foliar application [7,10]. Although the OTC ELISA kit is fast and sensitive, it is expensive and is not available in the market all the time.

Previous studies showed that europium can form a stable complex with tetracyclines and the fluorescence intensity can be boosted by the addition of a coligand [11]. The tetracyclines absorb the light at 388 nm and transfer it to europium which emits it as an intense and narrow band at 615 nm [11]. The europium method has been successfully used to detect several tetracyclines in different matrixes including meat, milk, serum, and urine [12,13]. Although the europium method is sensitive and has been known for more than twenty-five years, it has not been applied to plant tissues until 2021 [10].

In our previous study, we established a fluorometric method for the detection of OTC in citrus leaves by complexing it with europium and cetyltrimethylammonium chloride (CTAC) [10]. We also found that phenols could interfere with europium assay by reacting with the sensitizing reagent, CTAC [10]. To avoid this interference, we replaced the CTCA with Triton X-100. Unfortunately, Triton X-100 did not improve the fluorescence intensity of the Eu-OTC complex [10]. Consequently, we decided to clean the sample using solid-phase extraction. The solid-phase extraction (SPE) improved the fluorescence intensity and enhanced the recovery (75 ± 7.6%) of OTC [10]. The europium method was also used to determine the level of OTC in trunk-injected trees [10]. The levels of OTC obtained by the fluorometric method were close to those obtained by the ELISA method [10].

Previous results also showed that the europium-tetracycline (Eu-Tc) complex can be used to visualize citrate [14]. The fluorescence intensity of the europium-tetracycline-citrate (Eu-Tc-Cit) complex at 615-nm was significantly higher than that of Eu-Tc [14]. The stoichiometry of the Eu-Tc-Cit complex was 1:1:2 as measured by Job’s method [14]. It is believed that the citrate can chelate the Eu(III) through the oxygen atoms of the hydroxy and carboxy groups. Citrate chelation with Eu(III) enhances the fluorescence intensity of the Eu-Tc complex by displacing the water from the coordination sites of Eu(III) [14]. In our current study, we investigate the use of citrate instead of the toxic CTAC as a coligand for the detection of OTC in citrus tissues using the europium method. We also determined the optimal conditions for the formation of the Eu-OTC-Cit complex in tris buffer. The citrate is safer and cheaper than CTAC, and available in most labs. In addition, the citrate does not react with phenols as does CTAC.

The plant tissues are complex matrixes, which are rich in primary and secondary metabolites. Therefore, the detection of OTC in plant tissues is a challenge. Like other plant tissues, citrus leaves are rich in flavonoids, phenols, and other metabolites, which could interfere with the determination of OTC using the europium method. Thus, we studied the possible interference of citrus metabolites with the formation of Eu-OTC-Cit complex.

## 2. Results 

### 2.1. Optimization of the Europium Citrate Method

#### 2.1.1. Effect of pH

**Figure 1:**
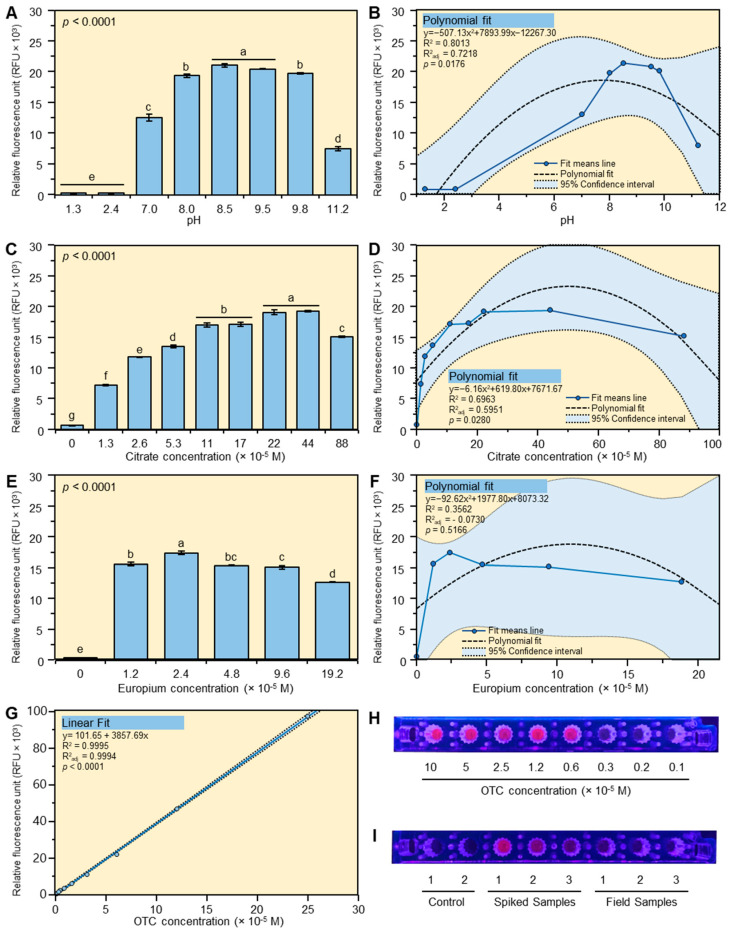
Optimization of the europium method using citrate as a sensitizing agent. Effect of pH presented as 2-D column (**A**) & polynomial fit (**B**), citrate presented as 2-D column (**C**) & polynomial fit (**D**), europium presented as 2-D column (**E**) & polynomial fit (**F**), and OTC (**G**) concentration on the fluorescence intensity of Eu-OTC-Cit complex. Data are the means ± SD of three replicates (*n* = 3). Columns with different letters are significantly different by Tukey HSD (*p* < 0.05). Fluorescence imaging of OTC in standard (**H**), spiked samples (**I**), and field samples (**I**). Spiked and field samples were extracted using 1 M HCl containing 2.2% trichloroacetic acid and cleaned using an HLB cartridge before being mixed with europium and citrate in tris buffer (pH 8.5). Samples were placed in a 96-well microplate and their image was taken under ultraviolet light (400 nm).

The effect of pH on the fluorescence intensity of the Eu-OTC-Cit complex was studied between pH 1.3 and pH 11.2. The fluorescence intensity of the Eu-OTC-Cit ternary complex was very low at low pH (1.3 and 2.4) and increased with pH (Figure 1A,B). The optimum fluorescence intensity of the Eu-OTC-Cit complex was observed between 8.5 and 9.5 pH and it declined thereafter (Figure 1A,B). 

#### 2.1.2. Effect of Citrate

The effect of citrate concentration on the fluorescence intensity of the Eu-OTC-Cit complex was studied between 0 and 88 × 10^−5^ M (Figure 1C,D). The fluorescence intensity of the Eu-OTC-Cit complex increased by increasing the citrate concentration from 0 to 44 × 10^−5^ M and declined thereafter (Figure 1C,D). The maximum fluorescence intensity was observed between 22 × 10^−5^ M and 44 × 10^−5^ M citrate in the final reaction mixture (Figure 1C,D). A significant decrease in fluorescence intensity was observed when the citrate concentration was increased from 44 × 10^−5^ M to 88 × 10^−5^ M. The previous results showed that the fluorescence intensity of the Eu-OTC complex was tremendously enhanced after the addition of citrate. 

#### 2.1.3. Effect of Europium

The effect of europium concentration on fluorescence intensity of the Eu-OTC-Cit complex is shown in Figure 1E,F. The fluorescence intensity was significantly enhanced by the addition of Eu(III) even at a very low concentration (1.2 × 10^−5^ M). The fluorescence intensity slightly increased by increasing the europium concentration from 1.2 × 10^−5^ M to 2.4 × 10^−5^ M and decreased thereafter (Figure 1E,F). 

#### 2.1.4. Linear Range for OTC

The effect of OTC concentration was studied between 0–100 ppm. The linear range of OTC was between 0 to 25 ppm under our experiment condition with a high coefficient of variation (R^2^: 0.9995) (Figure 1G). The fluorescence intensity above 25 ppm was very high and could not be measured by the fluorometer (overflow). We were also able to visualize the OTC in standards, spiked samples, and field samples under ultraviolet light (400 nm) after its complexation with Eu(III) and citrate (Figure 1H,I).

### 2.2. Interference of Plant Matrix

**Figure 2:**
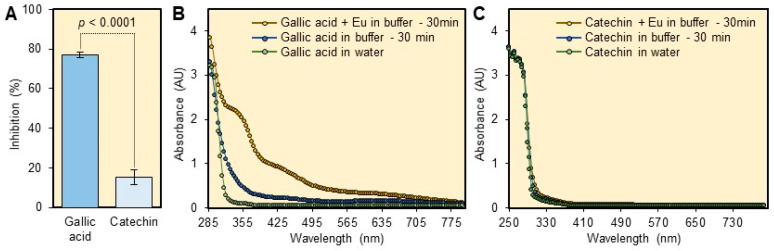
Interference of phenols and flavonoids with the europium method. (**A**) Relative fluorescence inhibition (%) of gallic acid and catechin when present at 100 ppm in the final assay mixture. Data are the means ± SD of five replicates (*n* = 5). Averages with *p*-values < 0.05 are significantly different using a two-tailed student *t*-test. (**B**) The UV-visible spectra of gallic acid after being incubated for 30 min in tris buffer (pH 8.5) with or without europium. (**C**) UV-visible spectra of catechin after being incubated for 30 min in tris buffer (pH 8.5) with or without europium.

Low fluorescence intensity was observed when the OTC standard was prepared in the sample matrix (data not shown). This result indicated that some plant metabolites interfere with the europium method. Therefore, we decided to study the interference of plant phenols and flavonoids. Gallic acid showed high inhibition (77.0 ± 1.4%) of the fluorescence intensity when it was present at 100 ppm in the reaction mixture (Figure 2A). Whereas, catechin showed a slight inhibition (15.1 ± 3.8%) of the fluorescence intensity of the Eu-OTC-Cit complex when presents at 100 ppm in the final assay mixture (Figure 2A). When gallic acid was mixed with the tris buffer and left at room temperature for 30 min it gave a light brown color. The UV-visible spectra of the degradation product are shown in Figure 2B. A similar but more intense color was also noticed after 30 min of mixing of gallic acid with Eu(III) in the tris buffer (Figure 2B). These results indicated that gallic acid degrades slowly at pH 8.5 and its degradation was enhanced in the presence of europium. The UV-visible spectra of catechin did not show a significant change after it was incubated in tris buffer in the presence of Eu(III) (Figure 2C). 

### 2.3. OTC Recovery from Spiked Citrus Leaves

**Figure 3:**
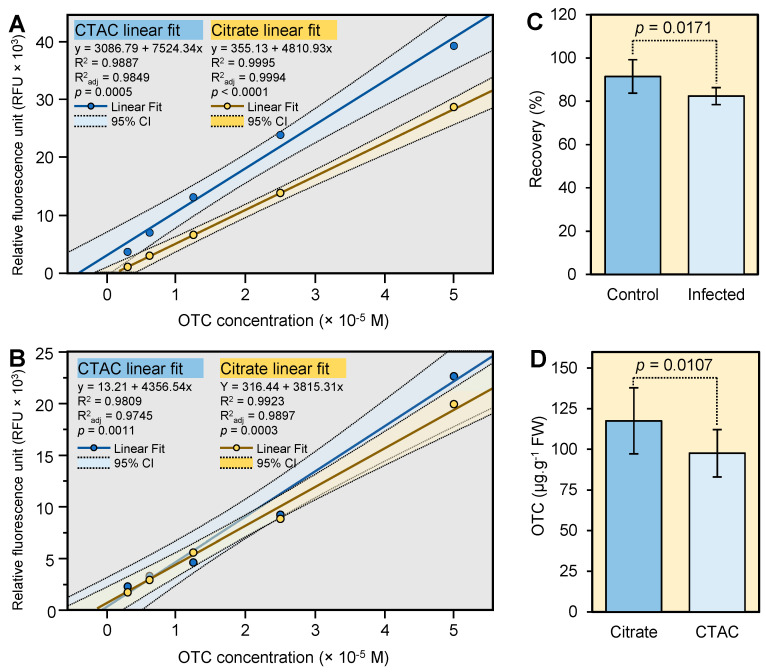
Recovery of OTC from spiked citrus leaf samples and application of the new method to field’s samples. (**A**) Standard curves of pure OTC prepared in 60% methanol using CTAC or citrate as a coligand. (**B**) Standard curves of OTC prepared in plant matrix (control samples extracted using 1 M HCl containing 2.2% trichloroacetic acid) and cleaned using an HLB cartridge. (**C**) Percentage recoveries of OTC from spiked healthy and *C*Las-infected citrus leaf samples as determined by the europium method using citrate as a sensitizing agent. (**D**) Levels of OTC in citrus leaves obtained from trunk-injected trees as determined by the europium method using citrate or CTAC as a sensitizing agent. Data are the means ± SD of five replicates (*n* = 5). Values with *p*-values < 0.05 are significantly different using a two-tailed student *t*-test.

To minimize the interference of plant metabolites with the europium method, the sample extract was cleaned using a hydrophilic-lipophilic balance (HLB) SPE cartridge. Two OTC standard curves were generated (Figure 3A,B). The first standard curve was prepared in 60% methanol and was measured directly. While the second standard curve was prepared in the sample matrix and cleaned using the HLB cartridge. The response of the pure OTC standard curves was higher than those prepared in the sample matrix and cleaned using the HLB cartridge (Figure 3A,B). 

The response of the pure OTC standard curve (prepared in 60% methanol) using CTAC was about two times higher than that generated using citrate (Figure 3A). However, the response of the OTC standard curve prepared in the sample matrix (cleaned using SPE) and measured using the CTAC method was similar to that measured using the citrate method (Figure 3A,B). The OTC recovery from spiked healthy and *C*Las-infected citrus leaves was 91.4 ± 7.8% and 82.4 ± 3.9%, respectively (Figure 3C). This result indicated that the developed method can be successfully used to estimate OTC levels in healthy and *C*Las-infected leaves. 

### 2.4. Application of the New Method to Field’s Samples

The new method was also used to estimate the level of OTC in leaves collected from trunk-injected trees. The level of OTC as measured using the current method (Eu-OTC-Cit) was 117.5 ± 20.3 µg g^−1^ FWT (Figure 3D). The level of OTC using the fluorescence method developed in our previous study (Eu-OTC-CTAC) was 97.5 ± 14.6 µg g^−1^ FWT (Figure 3D). This result indicated that the citrate can be successfully used to replace the CTAC. 

## 3. Discussion 

### 3.1. Optimization of the Method

Our result showed that pH 8.5 was the optimum pH for the formation of the Eu-OTC-Cit complex, whereas low (<2.4) and high pH (>11.2) showed very low fluorescence intensity. Similar luminescence intensities were reported for the Eu-OTC-Cit complex in urotorpin (hexamethylene tetramine)-HCl buffer [1]. Very low luminescence intensity (<25 a.u.) was observed at low pH (<5) and the intensity increased by increasing the pH [1]. The maximum luminescence was observed between pH 7.0 and pH 8, and it significantly decreased at pH 9.0 and above [1]. The low luminescence at low pH was explained by a low degree of complexation due to the protonation of the carboxy group of citrate and the endiolate group of OTC [1]. On the other hand, the low luminescence intensity at high pH (>11) was explained by the degradation of the Eu-OTC-Cit complex and the formation of europium hydroxide [1]. The maximum fluorescence intensity of the Eu-OTC-Cit complex in 10 mM N-(2-Hydroxyethyl)piperazine-N′-(2-ethanesulfonic acid) (HEPES) buffer was also found at pH 8.0 [14]. 

The optimum fluorescence intensity was observed when citrate was present at 22 × 10^−5^ M to 44 × 10^−5^ M in the reaction mixture. The optimum luminescence intensity for the determination of OTC in serum in urotorpin buffer (pH 7) was found at 1 × 10^−4^ M citrate [1]. The maximum fluorescence intensity was observed at 2.4 × 10^−5^ M europium. The optimum luminescence intensity of Eu-OTC-Cit complex in urotorpin buffer (pH 7) was also found at a very low concentration of europium (1 × 10^−4^ M) [1]. The linear range of OTC was between 0 to 25 ppm. A similar linear (0–23 ppm) range for OTC was reported using citrate as a coligand [1].

### 3.2. Interference of Plant Matrix

Plant tissues are complex matrixes and are rich in minerals and primary and secondary metabolites. Previous reports showed that OTC can complex with different cations including copper, zirconium, and iron [15]. Consequently, the detection of OTC in plant tissues is a challenge [10]. In our previous study, we showed that the citrus matrix significantly decreased the fluorescence intensity of the Eu-OTC-CTAC [10]. Yellow color was observed after mixing gallic acid with CTAC in tris buffer (pH 8.5), indicating a reaction between CTAC and gallic acid [10].

Significant inhibition of the fluorescence intensity was also observed in the current study when gallic acid was mixed with europium and citrate in tris buffer. Further investigation showed that gallic acid degrades rapidly at pH 8.5 in the presence of europium. Slow degradation of gallic acid was also observed in tris buffer without the addition of europium. In agreement with our current finding, a previous study showed that some phenolic compounds including gallic, caffeic, and chlorogenic acids, were not stable at high pH [16]. The degradation of gallic acid was enhanced by high pH, air, light, and by the presence of Fe^+3^ cation [17]. The previous results indicated that the presence of Eu^+3^ could enhance the degradation of gallic acid, by acting as an oxidizing agent at high pH. The oxidation products of phenols could decrease the fluorescence intensity by absorbing the applied and emitted light. In addition, the oxidation of phenols could also reduce the level of available Eu^+3^. The interference of the plant metabolites with the europium method indicated that OTC extract should be cleaned before being measured by the fluorometric method.

### 3.3. Recovery of OTC from Spiked Leaves

The recovery of OTC from spiked leaf samples was higher than 82%, indicating that the extraction and the cleanup procedures were efficient. Similar recovery (75 ± 7.6%) was reported for OTC from spiked citrus leaves using the Eu-OTC-CTAC complex [10]. The limit of detection (LOD) of OTC using our current method is about 6 µg g^−1^ FWT and it can be lowered to 3 µg g^−1^ FWT by eluting 0.5 ml of the sample extract into a 500-mg HLB cartridge [10]. 

### 3.4. Comparison between the Citrate and CTAC Method and Application of These Method to Field’s Samples

We used our current method to estimate the level of OTC in citrus leaves taken from trunk-injected trees and compared it to the CTAC method developed in our previous study [10]. The fluorescence intensity of the pure OTC standard curve generated using citrate was lower than that generated using CTAC, indicating that CTAC was a better sensitizing agent than citrate in pure solution. However, the fluorescence intensity of the OTC standard curve prepared in the sample matrix and measured using the CTAC method was similar to that measured using the citrate method. This result indicated that the reaction of phenols with the CTAC surfactant could also contribute to the inhibition of the fluorescence intensity besides the degradation of phenols at high pH in the presence of europium. The level of OTC using citrate as a sensitizing agent was similar to that obtained using CTAC. This result indicated that citrate can be successfully used to replace the CTAC surfactant. Our previous study also showed that the levels of OTC measured using the Eu-OTC-CTAC complex were similar to those measured using the ELISA kit [10].

OTC has a wide range of antimicrobial activity and high potency and it has been approved for the control of many plant diseases including Huanglongbing. Therefore, a sensitive and reliable method is crucial for the detection of OTC in plant tissues. The europium method has been successfully used for the detection of tetracyclines in different matrixes including blood, urine, serum, and meat. However, it has not been applied to plant tissues until our recent study [10]. In that study, we established a fluorometric method for the analysis of OTC in citrus leaves by complexing it with europium and CTAC [10]. In the current study, we replaced CTAC with citrate and optimized the europium method for the detection of OTC in citrus leaves. Our results showed that the sensitivity and the recovery of the citrate method were similar to that of the CTAC method. In addition, the EDTA is not required for the citrate method, which means fewer reagents. Furthermore, citrate has several advantages over CTAC: (1) it is safer and cheaper than CTAC, (2) it does not react with phenols as does CTAC, and (3) it is available in most labs. Our result shows that citrate was an excellent sensitizing agent and could be successfully used for the detection of OTC in citrus trees. The fluorometric method developed in this study based on the use of citrate could be another powerful tool to trace OTC in citrus plants. 

## 4. Material and Methods

### 4.1. Optimization of the Method

#### 4.1.1. Effect of pH

The tris buffer was adjusted with HCl or NaOH to different pH levels (1.3, 2.4, 7, 8, 8.5, 9.8, and 11.2) and the fluorescence assay was performed using 300 µL tris buffer (100 mM), 40 µL of 2 mM citrate, 20 µL europium chloride (1.25 mM), and 100 µL of 5 ppm OTC standard. After 30 min incubation in dark at room temperate, the fluorescence intensity was measured as described previously [10]. Three samples were analyzed at each pH. 

#### 4.1.2. Effect of Citrate

To study the effect of citrate on the fluorescence intensity of the Eu-OTC-Cit complex, different citrate solutions (10, 5, 2.5, 2, 1.2, 0.6, 0.3, 0.15, and 0 mM) were prepared in water. The fluorescence assay was conducted using 300 µL tris buffer (0.1 M pH, 8.5), 100 µL of 5 ppm OTC standard, 40 µL of citrate, and 20 µL of europium chloride (1.25 mM). Three samples were analyzed at each concentration.

#### 4.1.3. Effect of Europium

To study the effect of europium on the fluorescence intensity of Eu-OTC-Cit complex, different quantities (0, 5, 10, 20, 40, and 80 µL) of europium chloride (1.25 mM) was mixed with 300 µL tris buffer (0.1 M, pH 8.5), 100 µL of 5 ppm OTC standard, and 40 µL of 2 mM citrate and the final volume of was adjusted to 520 µL using water. Three samples were analyzed at each concentration.

#### 4.1.4. Effect of OTC

To examine the linear range of OTC standard, the fluorescence assay was conducted using 100 µL standard (100, 50, 25, 12.5, 6.2, 3.1, 1.5, 0.7, 0.3, 0.15, 0.05, and 0 ppm), 300 µL tris buffer (0.1 M, pH 8.5), 40 µL of 2 mM citrate, and 20 µL of europium chloride (1.25 mM).

### 4.2. Inhibition of Fluorescence by Plant Matrix

To test the effect of plant matrix on the fluorescence intensity of Eu-OTC-Cit complex, we initially tried to extract the OTC using diluted HCl (0.1 N HCl, 0.01% EDTA, pH adjusted to 4.0 using 1 N NaOH) as described in our previous study [10]. Because low fluorescence intensity was observed in the presence of the plant matrix, we decided to investigate the effect of plant metabolites (flavonoids and phenols) on the fluorescence intensity of the Eu-OTC-Cit complex. Gallic acid and catechin were chosen as a representative for phenols and flavonoids, respectively. Gallic acid and catechin are commonly used to estimate total phenols and flavonoids in plants [18].

To study the interference of gallic acid with the EU-OTC-Cit complex, a 100 ppm gallic acid or catechin in 5 ppm OTC standard were prepared as described previously [10]. The fluorescence assay was performed by mixing 300 µL of tris buffer, 40 µL of 2 mM citrate, 10 µL of europium solution, and 100 µL of gallic acid (100 ppm) or catechin (100 ppm) solution containing 5 ppm OTC. The % inhibition of catechin and gallic acid was calculated relative to pure OTC standard (5 ppm). Each treatment was measured five times.

To record the UV-Visible spectra for degradation product of gallic acid when mixed with europium in tris buffer, a 100-µL aliquot of 1000 ppm gallic was mixed with 300 µL of tris buffer and 50 µL of europium chloride (1.25 mM), and the mixture was left in dark at room temperature for 30 min. To study the degradation gallic acid in tris buffer alone, a 100-µL aliquot of 1000 ppm gallic was mixed with 300 µL of tris buffer and 50 µL of water, and the mixture was left in dark at room temperature for 30 min. To record the spectra of gallic acid, a 100-µL of 1000 ppm gallic was mixed with 350 µL of water. The previous procedures were repeated with catechin. The UV-vis was measured between 250–800 nm using Gen5 microplate reader (Biotek, Winooski, VT, USA).

### 4.3. Extraction of OTC from Spiked Leaves

Leaves were collected from two-year-old healthy (*n* = 5) or *C*Las-infected (*n* = 5) Valencia sweet orange (*Citrus sinensis* (L.) Osbeck). The *C*Las infection was confirmed by leaf symptoms and PCR analysis [9]. Three leaves were collected from each tree. Citrus leaves were ground in liquid nitrogen and a 100-mg aliquot of the ground tissues spiked with 50 µL of OTC standard (200 ppm) [10]. Ten samples (5 *C*Las-infected and 5 healthy) were spiked with OTC standard. The OTC was extracted using an acidic solution (1 M HCl, 2.2% trichloroacetic acid) as reported previously [10].

### 4.4. Solid-Phase Extraction (SPE)

The SPE was conducted using an Oasis HLB (Waters, Milford, MA, USA) cartridge (3 cc, 60 mg) cartridge as described previously [10]. A set of standards was prepared in the sample matrix (supernatant from control samples) and cleaned using SPE. Another set of standards was prepared in 60% methanol and analyzed directly. The recovery percentage was calculated using the standard curve that was prepared in the sample matrix and cleaned using the HLB cartridge.

### 4.5. Fluorescence Assay

The assay was performed using 100 µL standard or sample, 300 µL tris buffer (100 mM, pH 8.5), 40 uL of 2 mM citrate, and 15 uL of europium chloride (1.25 mM).

### 4.6. Application of the New Method to Field Samples

Trunk injection of OTC (1.7 g per tree) into five-year-old Hamlin trees (*n* = 5) was performed as described in our previous study [10]. OTC was extracted using a mixture of HCl and trichloroacetic acid, cleaned using an HLB cartridge, and analyzed using the fluorescent assay as described above. Also, the fluorescent assay was repeated using 1% Cetyltrimethylammonium chloride (CTAC) as described in our previous report [10].

### 4.7. Statistical Analysis

Data were analyzed using JMP Pro 15.0 software (SAS, Cary, NC, USA). The relative fluorescence inhibition (%) of catechin was compared to that of gallic acid using a two-tailed *t*-test (*p* < 0.05). The average OTC recovery from control and *C*Las-infected leaves were compared to each other using a two-tailed *t*-test (*p* < 0.05). The level of OTC measured by the citrate method was also compared with that measured by the CTAC method using a two-tailed t-*t*est (*p* < 0.05). The pairwise comparison using the Tukey-Kramer honestly significant difference test (Tukey HSD) was used to compare levels of the relative fluorescence intensity at different pHs, citrate, and europium concentrations.

## 5. Conclusions

The fluorescence intensity of the Eu-OTC complex was significantly enhanced after the addition of citrate, indicating that the citrate was an excellent sensitizing agent. The use of citrate as a coligand also enabled us to visualize and measure OTC in the sample extract of citrus leaves. A high recovery (>82%) of OTC from spiked healthy and *C*Las-infected leaves was obtained when citrate was used as a coligand. We also showed that the europium-citrate method can be effectively used to measure the level of OTC in trunk-injected citrus. The levels of OTC in trunk-injected trees measured using the europium-citrate method was similar to that obtained using the europium-CTAC method. In addition, replacement of CTAC with citrate could minimize the risks of the hazards in the workplace and environment. The development of sensitive and reliable methods for the detection of OTC in citrus plants could enhance the use of OTC for the control of the Huanglongbing disease.

## Data Availability

Data is contained within the article.
